# HPV-51 or HPV-52 Infection Could Impair Sperm Quality in Infertile Patients: A Preliminary Study on Our Experience from North-Western Italy

**DOI:** 10.3390/tropicalmed10020036

**Published:** 2025-01-28

**Authors:** Claudia Omes, Mariangela Rienzi, Roberta Rossini, Manuela Piccinino, Rossella Elena Nappi

**Affiliations:** 1 Center for Reproductive Medicine—Obstetrics and Gynecology Unit 2, Woman and Child Health Department, Fondazione IRCCS Policlinico San Matteo, 27100 Pavia, Italy; r.rossini@smatteo.pv.it (R.R.); m.piccinino@smatteo.pv.it (M.P.); r.nappi@smatteo.pv.it (R.E.N.); 2 Department of Clinical, Surgical, Diagnostic and Pediatric Sciences, University of Pavia, 27100 Pavia, Italy; mariangela.rienzi@unipv.it

**Keywords:** HPV, semen, sperm motility, male infertility, sperm quality, cancer, prevention

## Abstract

Human papillomavirus (HPV) infection is one of the most common sexually transmitted infections in all genders worldwide. Its association with male infertility is deeply investigated, although there are conflicting data on the role of the virus in the impairment of semen quality and reduced reproductive outcomes. In this study, we considered 335 semen samples of males (age: 37.63 ± 6.02 years) belonging to infertile couples who did not conceive a pregnancy after 12 months of unprotected intercourse. Residual semen samples, after routine sperm analysis, were used to amplify and type viral DNA. Positive or negative HPV semen samples were compared. In total, 42.51% (139/327) were positive for at least one HPV genotype, and in 54.68% (76/139), positivity was due to a high-risk (HR) genotype. The most prevalent was HPV-16 (16.55%) followed by HPV-52 (10.07%) and HPV-51 (7.91%). Overall, no significant differences emerged in terms of sperm concentration, sperm motility, and morphology between the two groups. However, a considerable reduction in sperm motility was found in the presence of HPV-51 or HPV-52. These data point to the importance of HPV screening in semen analysis to evaluate patients that might have a higher risk of infertility according to the type of HPV genotype.

## 1. Introduction

In recent years, there has been growing interest in the relationship between male HPV infection and infertility [1]. It seems also crucial to further understand HPV prevalence in men because of its impact on cervical cancer in women [2]. HPV is usually sexually transmitted, and 4.5% of all cancers in the world are attributable to HPV, with a higher prevalence in women (8.6%) than in men (0.8%) [3]. The lower prevalence of cancer in the male genital tract is probably due to the more rapid viral clearance (5.9 months) [4], despite the infection occurring in 31% of sexually active men over 15 years of age [2]. These results are consistent with considering men as a reservoir for HPV infection [2] and highlight the importance of including men in vaccination and prevention campaigns, implemented to reduce the spread of the virus and fight related cancers. While the causality between the persistence of the HPV infection and the onset of cervical tumors is clear, the impact that this infection has on the quality of seminal fluid and the outcome of in vitro fertilization procedures is still controversial. Many studies in the literature showed a higher prevalence of HPV semen infection in men affected by idiopathic infertility (range 10–35.7%) compared to the global population (range 2–31%) [1,5,6,7,8,9]. Some evidence demonstrated an impairment of semen quality, especially in sperm motility, with a significant decrease in progressive motility in HPV-positive patients compared to HPV-negative ones [5,10,11,12]. However, this correlation remains controversial because other studies found no differences between these two groups [13,14,15]. One of the possible explanations for conflicting results in the literature could be due to the different impact that a specific genotype might have compared to another. By comparing the sperm quality of semen infected by HR-HPV (High-Risk HPV) or LR-HPV (Low-Risk HPV), there was evidence that HR-HPV genotypes have a greater impact on worsening semen quality [16,17] and increase DNA fragmentation [14,18].

Regardless of the effects that the infection may have on the quality of the seminal fluid, it remains to be understood whether its presence can affect obtaining pregnancy, especially during in vitro fertilization procedures. It was reported that HPV sperm infection was higher in couples with recurrent pregnancy loss (RPL) than in fertile ones [17,19]. This evidence was also confirmed in couples undergoing IVF procedures [20] and in those with a higher miscarriage rate (MR) [10,19,21].

The pathogenic mechanism used by the virus to affect human sperm is not yet fully known. In vitro studies showed the presence of HPV on the head of sperm in a sub-group of patients infected by the virus [22]. For these reasons, the same authors suggested investigating the link between sperm cells and HPV by a FISH test [17]. If the virus was bound, they suggested using a modified swim-up with added hyaluronidase in order to remove the binding during IVF procedures [23]. Another study showed a co-localization between HPV-16 and aquaporin-8 proteins (AQP8) in the mid-piece of the spermatozoa [24]. Although there are no conclusive data, this evidence seems to argue in favor of a possible role of the infection in reducing the functionality of the antioxidant systems of the spermatozoa mediated by aquaporin-8.

In humans, it is difficult to study whether the virus can also be transferred from sperm to embryos. In the animal model, HPV DNA transmission to the early mouse embryos after fertilizing oocytes with spermatozoa pre-incubated with plasmid vectors containing HPV-16 and -18 has been demonstrated [25].

Our present study had the aim to investigate the prevalence of HPV infection in men undergoing diagnostic fertility investigation in a basin located in North-Western Italy. We analyzed the prevalence of HPV infection, the distribution of HPV genotypes, and sperm parameters in the same semen samples.

## 2. Materials and Methods

### 2.1. Study Design and Samples

This retrospective study considered the sperm characteristics and HPV tests of 335 semen samples during routine clinical use. Semen samples were obtained by masturbation, after 2–7 days of sexual abstinence, and collected in a sterile container at the laboratory of the Center for Reproductive Medicine (Fondazione IRCCS Policlinico San Matteo, Pavia, Italy). The study was conducted according to the guidelines of the Ethics Committee of IRCCS San Matteo Foundation for observational data. Written informed consent was obtained from each subject involved in the study for the use of personal data.

### 2.2. Semen Analysis

Routine semen analysis was performed within 1 h of collection, according to the methods described by the 6th manual of the World Health Organization (WHO) [26], after 30 min of incubation at +37 °C. First, macroscopic analysis is carried out to assess volume, pH, fluidification, and viscosity.

Each sample was analyzed to determine sperm count, sperm motility, and kinematics of movement using a disposable counting chamber (Counting Chamber Makler, Sefi Medical Instruments, Haifa, Israel). We determined the spermatozoa concentration by using the chamber’s grid. The number of spermatozoa counted in any strip of 10 squares of the grid indicated their concentration in millions/mL. We counted at least three strips and the mean value was considered. The chamber has a depth of 10 µm that eliminates blurring and allows sperm to move freely. The system for grading motility was based on the distinction of spermatozoa with progressive (PR) or non-progressive (NP) motility and those that are immotile (IM), as reported by the WHO manual [26].

To evaluate sperm morphology, Diff-Quik-stained slides (Test Simplets, Origio, Denmark) were used. As indicated by the WHO manual [26], restricted criteria by Kruger to analyze at least 200 spermatozoa per sample were applied.

### 2.3. HPV Test

To detect and type HPV in semen fluid, DNA extraction was performed on samples (100–300 µL) using an automatic instrument (Maxwell MDX16, Promega Italia srl, Milan, Italy) based on paramagnetic particles. PCR of HPV sequences was performed from 10 µL of the solution added to 40 µL of AMP MIX (INNO-LiPA HPV Genotyping Extra II, Fujirebio Italia S.r.l., Roma, Italy) to amplify the L1 region using SPF10 primers (65 bp) for 40 cycles.

Positive and negative controls and blank reagents were introduced in each set of amplification. Simultaneously, amplification of the human HLA-DPB1 gene was included in the assay, as the internal control for DNA adequacy for all samples tested.

Our assay allows us to detect 13 HR-HPV genotypes (16, 18, 31, 33, 35, 39, 45, 51, 52, 56, 58, 59, 68), 6 intermediate or probable HR types (26, 53, 66, 70, 73 and 82), 9 LR-HPV types (6, 11, 40, 42, 43, 44, 54, 61 and 81), and 4 undetermined risk (UR) types (62, 67, 83, and 89) [27]. The test also detects positivity when an HPV genotype different from the 32 amplified from the kit infects the sample analyzed. These positive samples were identified as unclassified (NC).

### 2.4. Data Collection and Statistical Analysis

Data were collected regarding patient’s history, sperm characteristics, and HPV test results. Data on volume, sperm count, sperm motility, and sperm morphology were reported as mean and standard deviation (SD), and normal distribution was calculated (Shapiro–Wilk test).

Student t-tests, Mann–Whitney tests, or Kruskal–Wallis tests were used to analyze differences among the groups as appropriate. The chi-square test was used to study differences in HPV risk in the groups analyzed. A *p*-value ≤ 0.05 was considered statistically significant. All tests were two-sided. Data analysis was performed with GraphPad Prism 8.3.0 (San Diego, CA, USA).

## 3. Results

### 3.1. Samples

**Figure 1:**
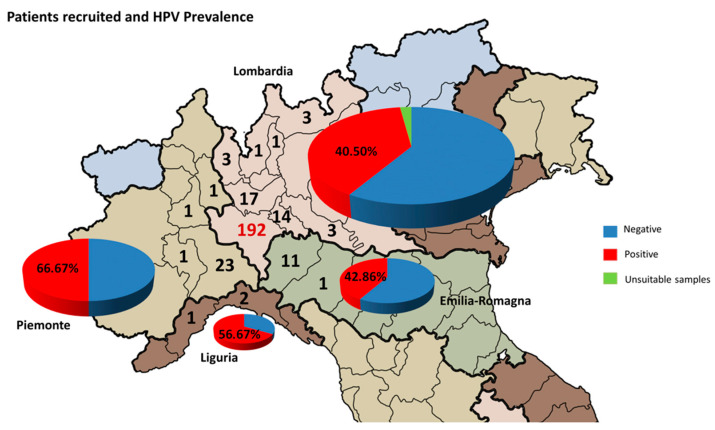
Geographic distribution of patients recruited for semen analysis and HPV test. Numbers in the map represent the patients recruited in each province within the region. The pie charts represent the percentage of positive samples for HPV infection (blue: negative; red: positive; green: unsuitable samples).

**Figure 2:**
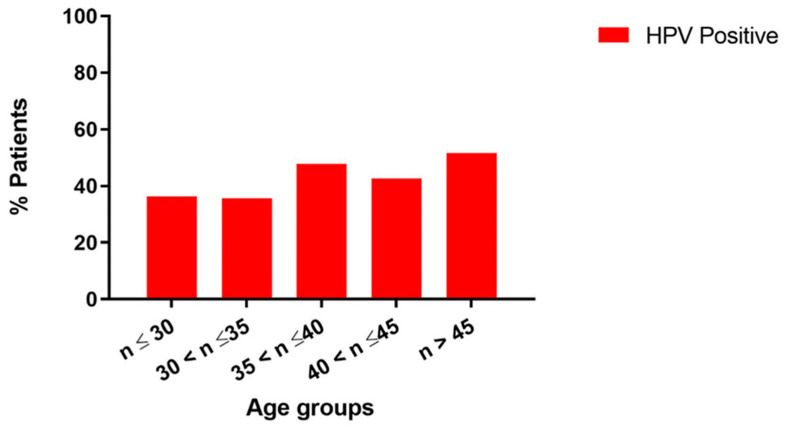
Histograms of the prevalence of HPV infection by age. Each histogram represents the percentage of semen samples positive for HPV infection divided by age groups.

In this study, we reported data on 335 semen samples from men (mean ± SD: 37.63 ± 6.02 years) undergoing routine diagnostic semen analysis for couples’ infertility management. Eight samples were discarded because they were unsuitable (absence of DNA molecules to amplify). The geographic distribution of our study sample and the prevalence of HPV infection are represented in Figure 1. In total, 42.51% (139/327) of the samples recruited were positive for at least one HPV genotype. The prevalence by age group was also analyzed. As reported in Figure 2, the prevalence of HPV infection showed a trend in favor of an age-related increase in the risk of infection even if it was not statistically significant (chi-square > 0.05). Indeed, by comparing age between negative and positive samples, we observed a higher age in the group affected by HPV infection (38.38 ± 5.96 vs. 37.13 ± 6.01 years; *p*-value < 0.05).

### 3.2. Semen Analysis and HPV Infection

Data on collected samples were divided into two groups in order to compare basal semen parameters between positive and negative samples for HPV infection. There were no statistically significant differences between negative and positive samples in terms of semen volume, sperm concentration, sperm motility (progressive, non-progressive, and total), and sperm morphology (Table 1). Then, the impact of the HPV genotype was investigated.

### 3.3. HPV Genotyping Distribution

**Figure 3:**
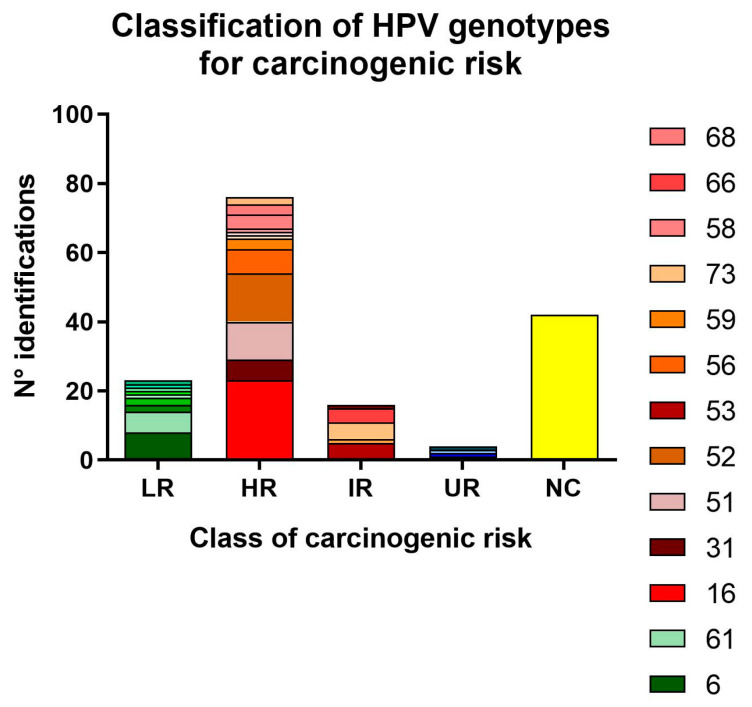
Histograms of the carcinogenic risk of HPV genotype. The histogram represents the number of semen samples positive for each HPV genotype grouped for the class of carcinogenic risk: LR for low risk, HR for high risk, IR for intermediate risk or probably HR, UR for undetermined risk, and NC for not classified genotyping.

HPV genotyping showed a higher prevalence of HR-HPV with 55.40% (77/139) of positivity due to a high-risk (HR) genotype, which represented 23.55% (77/327) of the entire study sample (Figure 3). In total, 12.23% (17/139) of positive patients showed at least one LR-HPV genotype without co-infection with HR-HPV. The most prevalent LR-HPV is HPV-6 (8/139; 5.76%) followed by HPV-61 (6/139; 4.32%). In 30.94% (43/139) of positive cases, the test used for HPV genotyping did not reveal the HPV genotype. For this reason, we were not able to establish the class of risk in these patients. The most prevalent HR-HPV is HPV-16 (23/139; 16.55% of positive samples), followed by HPV-52 (10.07%, 14/139) and HPV-51 (7.91%, 11/139). Semen parameters in negative patients and in those who were infected by HR or LR HPV genotypes were investigated but no statistical differences emerged (Table S1).

### 3.4. Impact of HPV Genotype on Semen Quality

**Figure 4:**
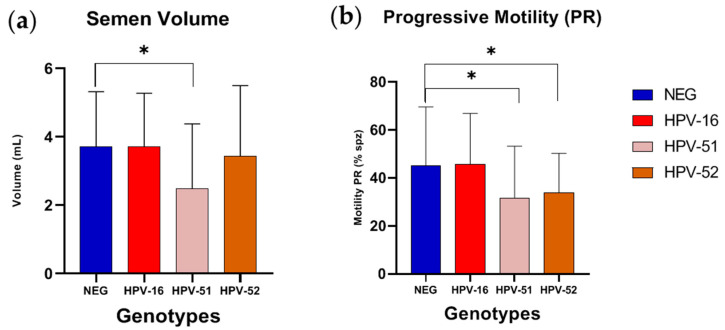
Comparison of parameters of negative and positive samples for the most prevalent HR-HPV. Histograms represent mean ± standard deviation of different parameters in negative samples and in those infected by HPV-16, HPV-51 and HPV-52 for (**a**) semen volume and (**b**) progressive motility. * Mann–Whitney test: *p*-value < 0.05.

The most prevalent HR-HPV genotypes were also investigated to determine their possible association with semen quality. Age and each semen parameters (volume, sperm concentration, motility, and morphology) were compared between negative and positive samples for HPV-16, HPV-51, and HPV-52. HPV-51 was associated with a decrease in sperm volume (3.72 ± 1.60 mL vs. 2.49 ± 1.89 mL, *p* = 0.0108) and progressive motility reduction (45.23 ± 24.39% vs. 31.91 ± 22.67%, *p* = 0.0230). Even HPV-52 was associated with a significant decrease in progressive motility (45.23 ± 24.39% vs. 33.93 ± 16.39%, *p* = 0.0344) (Figure 4, Table S2). There were no statistical differences among the other parameters analyzed (Figure S1).

## 4. Discussion

The extreme spread of HPV infection around the world has long brought attention to the impact that this infection has on the onset of tumors but also on its possible effect on human reproduction, being generally associated with ano-genital tract infections [5]. Recent findings underlined a possible correlation between HPV sperm infection and asthenozoospermia or idiopathic infertility [2,6,14,22,28]. HR-HPV in particular was associated with the impairment of sperm progressive motility [29], but studies about the impact of a specific HPV genotype on semen quality are hard to find.

Our findings showed a high positivity of semen samples of infertile men (42.51%), with no differences in terms of sperm parameters between negative or positive semen samples for HPV, but with a prevalence of HPV infection higher in respect to that generally estimated among men (31%) [2]. However, when a specific HR-HPV was considered, a decrease in sperm motility or semen volume was seen for some HR-HPVs, especially HPV-51 or HPV-52. On the other hand, HPV-16 did not seem to have an impact on semen quality. The differences observed in terms of sperm motility and semen volume dependent on HR-HPV genotype suggest that the effect on semen was probably due to the genotype and not to the presence of the virus itself. A recent study, for example, revealed that HR-HPV+ individuals showed higher levels of sperm necrosis and an increase in ROS (reactive oxygen species) compared to LR-HPV+ or negative control [30].

Considering the viral genotype, the most frequently detected genotypes in European men collected in a wider meta-analysis were HPV-16, HPV-51 and HPV-6, with HPV-52 ranking sixth [2]. Another recent study found HPV-42, HPV-16, HPV-53 and HPV-51 as the most prevalent [31]. The discrepancy of genotype distribution is probably due to the different prevalence in each local area [32]. Although there are several studies evaluating the quality of seminal fluid in the presence of HPV in general or in particular with HPV-16, there are no studies in the literature investigating the impact of HPV-51 and HPV-52 on semen parameters individually.

That being so, it is of utmost importance to screen HPV in semen analysis with the aim to identify those patients at higher risk of infertility. It is likely that the heterogeneity of data reported in the literature might depend on how patients with HPV infection are classified or on the sensitivity of the tests used for genotyping.

Although we did not investigate the effect of HPV infection on reproductive outcomes, it has been known that a decrease of sperm progressive motility and semen volume (which results in a reduction in total sperm count), might exert an impact on men’s fertility and therefore on the possibility of achieving pregnancy [26].

The data reported here strongly support the importance of HPV screening in partners of women attempting to achieve pregnancy because semen quality could be further improved following virus eradication. There is actually no effective therapy to treat HPV infection. Prophylactic vaccination was introduced for children before sexual debut, but there are now campaigns for the immunization of adults to prevent recurrences in patients treated for cervical lesions or recurrent respiratory papillomatosis [33]. The nonavalent vaccine, which is the one most commonly used at present, is characterized by virus-like particles (VLPs) of HPV-16, -18, -6, -11, -31, -33, -45, -52 and 58. Two of the most represented HPV genotypes in the population analyzed in this study would be covered by the nonavalent vaccine. This vaccine has excellent efficacy in protecting from incident HPV infection [33,34] and prevents cancer progression and recurrence in cases of HPV-related disease [35]. For this reason, it is now used more and more widely as a strategy to stimulate the immune system to produce neutralizing antibodies that prevent the attachment of the virus to the basement membrane and help neutrophils to eliminate the antibody–virus complex, especially in women who have a diagnosis of cervical infection [35]. The wider spread of infection in men, as well as the persistence and increase in incidence as they get older, suggests the great importance of vaccination in all genders [2] to confine the diffusion of the infection and the burden of HPV-related cancer, but also to prevent the risk of infertility [2].

Nowadays, the management of HPV infection in infertile couples is not fully shared among experts in the field. The available literature recommends engaging in protected sexual intercourse to facilitate viral clearance. In addition, it is advised to test the link of HPV with sperm by using the FISH test in order to decide whether to apply the modified swim-up with hyaluronidase to remove the linked virus [5,23]. As a good practice, it can be suggested to screen infertile men for HPV infection and vaccinate positive individuals in order to stimulate the immune system to eliminate the virus. Further studies will be necessary to corroborate our data due to the small number of our positive samples in order to evaluate the best practices for the effective management of HPV infection, especially in infertile couples.

## 5. Conclusions

The present study showed that a significant proportion (42.51%) of semen analyzed from infertile patients living in North-Western Italy were positive for at least one HPV genotype with a higher prevalence of HPV-16, followed by HPV-52 and HPV-51. No significant differences emerged in terms of sperm parameters between negative and positive groups, but when HPV-51 or HPV-52 were present, it seems that an impairment of semen quality can be identified. Overall, our data underline the importance of HPV screening in semen analysis to evaluate patients that have a higher risk of infertility associated with specific HPV genotypes.

## Figures and Tables

**Table 1:** Semen parameters of infertile men.

	Patients (*n* = 327)		
	Negative (*n* = 188)	Positive (*n* = 139)	*p*-Value (Mann–Whitney)
Age (years)	37.13 ± 6.01	38.38 ± 5.96	0.0494 *
Semen volume (mL)	3.72 ± 1.60	3.64 ± 1.48	0.7556
Sperm concentration (10^6^ spz ^a^/mL)	58.09 ± 54.97	62.44 ± 55.61	0.3758
Progressive motility (% PR)	45.23 ± 24.39	43.98 ± 20.03	0.7439
Non-progressive motility (% NP)	9.48 ± 6.74	8.92 ± 5.10	0.7841
Total Motility (% PR + NP)	54.72 ± 26.58	52.90 ± 20.08	0.6473
Morphology (% Normal)	2.21 ± 2.09	2.37 ± 2.43	0.5033

Value are mean ± SD; * Mann–Whitney test: *p*-value < 0.05; ^a^ spz = spermatozoa.

## Data Availability

The data presented in this study are available on request to the corresponding author.

## Supplementary Materials

The following supporting information can be downloaded at https://www.mdpi.com/article/10.3390/tropicalmed10020036/s1: Figure S1: Semen parameters of men distributed for class of carcinogenic risk of HPV genotype; Table S1: Comparison of parameters of negative and positive samples for the most prevalent HR-HPV genotype; Table S2: Comparison between semen parameters negative for HPV or positive for HPV-16. HPV-51. and HPV-52.
