# Supplementary material for: HPV-51 or HPV-52 Infection Could Impair Sperm Quality in Infertile Patients: A Preliminary Study on Our Experience from North-Western Italy
Source: Trop Med Infect Dis. 2025 Jan 28;10(2):36. doi: 10.3390/tropicalmed10020036 (PMC11861865; doi:10.3390/tropicalmed10020036)
Supplement: Supplementary file 1 [file tropicalmed-10-00036-s001.zip › tropicalmed-3261942-supplementary.pdf]

Table S1. Semen parameters distributed for class of carcinogenic risk of the HPV genotype.

	Patients (N=282)			
	**Negative (N=188)**	**HR (N= 77)**	**LR (N= 17)**	**P-value (Kruskal-Wallis test)**
**Age (years)**	37.13±6.01	37.79±5.75	36.88±5.84	0.2281
**Semen volume (ml)**	3.72±1.60	3.55±1.60	3.72±1.49	0.7815
**Sperm concentration (10^6 spz/ml)**	58.09±54.97	64.28±56.69	68.83±60.37	0.4670
**Progressive motility (% PR)**	45.23±24.39	45.13±19.52	53.00±18.17	0.2268
**Non-progressive motility (% NP)**	9.48±6.74	8.83±5.15	7.71±4.74	0.4852
**Total Motility (% PR+NP)**	54.72±26.58	53.96±19.09	60.71±17.27	0.3579
**Morphology (% Normal)**	2.21±2.09	2.55±2.77	2.41±2.12	0.7885

Value are Mean ± SD

Table S2. Comparison between semen parameters negative for HPV or positive for HPV-16. HPV-51. and HPV-52.

	Patients (N=236)				
	**Negative (N=188)**	**HPV-16 (N= 23)**	**HPV-51 (N= 11)**	**HPV-52 (N=14)**	**P-value (Kruskal-Wallis test)**
**Age (years)**	37.13±6.01	37.52±4.91	38.18±6.31	39.14±6.27	0.2825
**Semen volume (ml)**	3.72±1.60	3.72±1.56	2.49±1.89	3.44±2.06	0.0832
**Sperm concentration (10^6 spz/ml)**	58.09±54.97	74.19±60.85	50.42±30.64	51.35±42.35	0.5205
**Progressive motility (% PR)**	45.23±24.39	45.83±21.17	31.91±22.67	33.93±16.39	0.0257*
**Non-progressive motility (% NP)**	9.48±6.74	8.30±4.11	8.18±6.27	9.93±5.62	0.7799
**Total Motility (% PR+NP)**	54.72±26.58	54.13±20.02	40.09±22.83	45.43±18.08	0.1457
**Morphology (% Normal)**	2.21±2.09	3.22±4.03	1.36±1.29	1.79±1.48	0.3017

Value are Mean ± SD. *p-value<0.05

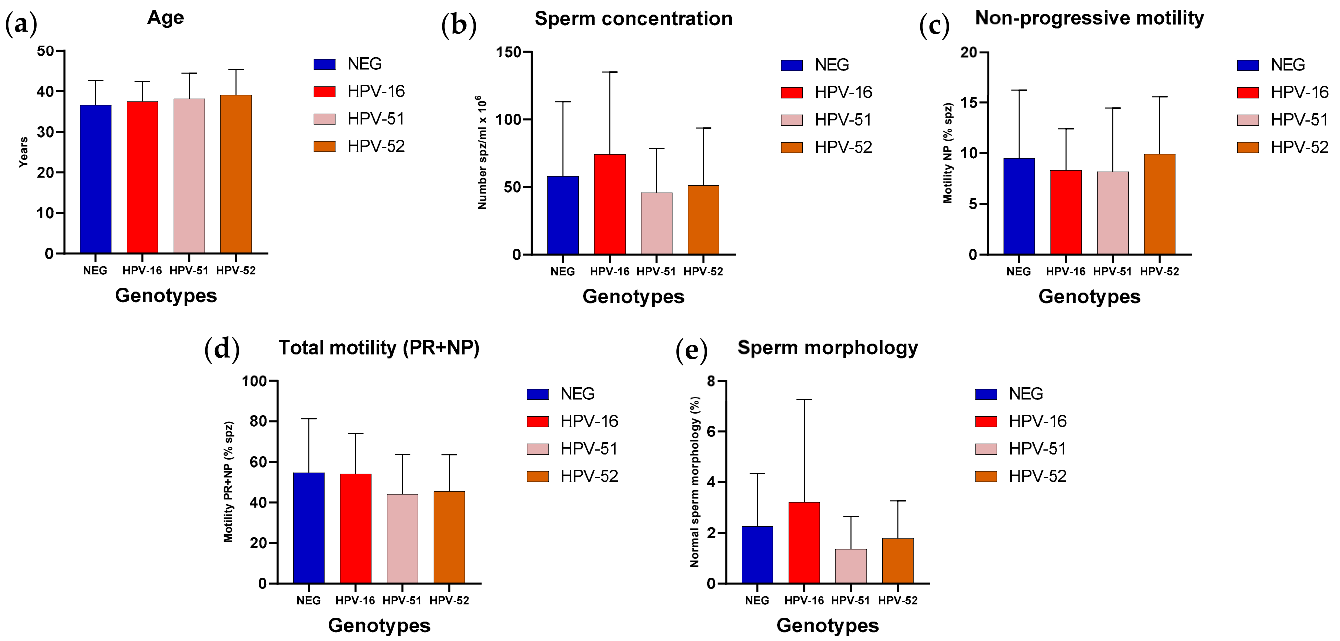

Figure S1. Comparison of parameters in negative samples and in samples positive for the most prevalent HR-HPV. Histograms represent media ± standard deviation of different parameters in negative samples and in those infected by HPV-16, HPV-51, and HPV-52: (a) age; (b) sperm concentration; (c) non-progressive motility; (d) total motility and (e) sperm morphology.
